# Shexiang Baoxin Pill, a Traditional Chinese Herbal Formula, Rescues the Cognitive Impairments in APP/PS1 Transgenic Mice

**DOI:** 10.3389/fphar.2020.01045

**Published:** 2020-07-14

**Authors:** Wei-Hui Hu, Shing-Hung Mak, Zhong-Yu Zheng, Ying-Jie Xia, Miranda Li Xu, Ran Duan, Tina Ting-Xia Dong, Shao-Ping Li, Chang-Sen Zhan, Xiao-Hui Shang, Karl Wah-Keung Tsim

**Affiliations:** ^1^ Shenzhen Key Laboratory of Edible and Medicinal Bioresources, HKUST Shenzhen Research Institute, Shenzhen, China; ^2^ Division of Life Science and Center for Chinese Medicine and State Key Laboratory of Molecular Neuroscience, The Hong Kong University of Science and Technology, Hong Kong, Hong Kong; ^3^ Joint Laboratory of Guangdong Province and Hong Kong Region on Marine Bioresource Conservation and Exploitation, College of Marine Sciences, South China Agricultural University, Guangzhou, China; ^4^ Institute of Chinese Medical Sciences, University of Macau, Macau, Macau; ^5^ Shanghai Engineering Research Center for Innovation of Solid Preparation of TCM, Shanghai, China; ^6^ Shanghai Hutchison Pharmaceuticals Ltd., Shanghai, China

**Keywords:** Shexiang Baoxin Pill, Chinese medicine, acetylcholinesterase, neuroprotective, cognitive enhancing effect, Alzheimer’s disease

## Abstract

**Background:**

Shexiang Baoxin Pill (SBP), a formulated traditional Chinese medicine (TCM), has been widely used to treat cardiovascular diseases for years. This herbal mixture has been shown to promote differentiation of cultured neuronal cells. Here, we aimed to investigate the effects of SBP in attenuating cognitive impairment in APP/PS1 transgenic mice.

**Methods:**

Ethanol and water extracts of SBP, denoted as SBP_EtOH_ and SBP_water_, were standardized and applied onto cultured rat pheochromocytoma PC12 cells. The potential effect of SBP_EtOH_ extract in attenuating the cognitive impairments in APP/PS1 transgenic mice was shown by following lines of evidence: (i) inhibition of Aβ fibril formation, (ii) suppression of secretions of cytokines, and (iii) improvement of behavioral tests by Morris water maze.

**Results:**

SBP_water_ and SBP_EtOH_ inhibited the formation of β-amyloid fibrils and protected the Aβ-induced cytotoxicity in cultured PC12 cells. In APP/PS1 transgenic mice, the treatment of SBP_EtOH_ inhibited expressions of NO, NOS, AChE, as well as aggregation of Aβ. Besides, the levels of pro-inflammatory cytokines were suppressed by SBP treatment in the transgenic mice. Importantly, the behavioral tests by Morris Water maze indicated that SBP attenuated cognitive impairments in APP/PS1 transgenic mice.

**Conclusion:**

The current result has supported the notion that SPB might ameliorate the cognitive impairment through multiple targets, suggesting that SBP could be considered as a promising anti-AD agent.

## Introduction 

Alzheimer’s disease (AD), characterized by dramatically declined of learning and memory, is the most common form of dementia affecting elderly population (Desgranges et al., 1998; Francis et al., 1999). The exact causes of AD remain unclear; however, the role of β-amyloid (Aβ) in the brain has been proposed to play key role in the pathological progress (Guidi et al., 2006). Fibrillar aggregate of Aβ is main constituent of plaques being found in AD patient’s brain, and therefore which has been considered as one of important causative events during AD pathogenesis (Chen et al., 2000; Goldsworthy and Vallence, 2013; Hampel, 2013). Under this scenario, drugs having inhibitory effects on formation of Aβ fibrils and Aβ fibril-mediated neurotoxicity, therefore, may possess therapeutic values for AD.

Traditional Chinese medicine (TCM) has been extensively used for treatment of various diseases in Asian countries for thousand years. Having a long historical usage, TCM has been proved to be effective and safe, especially in treating chronic diseases associated with aging (Gao et al., 2008; Yang et al., 2011). **S**hexiang **B**aoxin **P**ill (SBP), a herbal formula deriving from TCM theory, has been widely used for treatment of cardiovascular problems for many years (Yan et al., 2006). There are seven medicinal constituents in SBP, i.e. Moschus, Bovis Calculus Artifactus, Ginseng Radix et Rhizoma, Styrax, Cinnamomi Cortex, Borneolum Syntheticum, and Bufonis Venenum. SBP is being recorded in 2015 edition of Chinese Pharmacopoeia. Being on the market for years, the efficacy of SBP is well recognized for its rapid and effective action in resolving cardiovascular problems (Lu et al., 2018). In addition, several lines of evidence have indicated that SBP is effective to treat stable angina pectoris and chest pain, as caused by coronary heart disease (Hong et al., 2006; Yan et al., 2006; He et al., 2009; Tian et al., 2011). Besides, the potential therapeutic effects of SBP in treating other diseases, including early hypertensive renal injury and mental depression have been proposed (Chen et al., 2008; Zhao et al., 2018).

In neuronal culture, the application of SBP was shown to promote differentiation as well as the expressions of those neuronal biomarkers, and this action was mediated by activating cAMP/PKA protein kinase A (PKA) and cAMP responsive element binding protein (CREB) signaling pathways (Xu et al., 2019). This *in vivo* study therefore suggested the possible application of SBP in treating neurological disorders. Here, we are aiming to further extend the anti-AD function of SBP by using mouse model. The APP/PS1 transgenic mouse, a commonly used mouse model for AD analysis, was treated with the intake of SBP, and the behavioral and biochemical changes in these mice were determined.

## Materials and Methods

### Chemical and Medicinal Materials

A single batch of SBP and seven herbal materials or extracts were used here, i.e. Ginseng Radix et Rhizoma (supplied as the dried 75% ethanol extract of *Panax ginseng* root and rhizome, having more than 0.27% ginsenoside Re and ginsenoside Rg_1_ and ginsenoside Rb_1_ not less than 0.18% by weight, as defined by Chinese Pharmacopoeia; voucher #190603), Moschus (the dried secretion of musk sac of adult male *Moschus berezovskii*, *M. sifanicus*, or *M. moschiferus;* muscone is the main ingredient in Moschus; voucher #2017YR072), Cinnamomi Cortex (the dried stem bark of *Cinnamomum cassia*; voucher #180601), Bovis Calculus Artifactus (prepared with powder of cholic acid, cow bile, taurine, bilirubin, cholesterol, hyodeoxycholic acid, and trace elements; voucher #180511), Styrax (the acaroid resin obtained from the trunk of *Liquidambar orientalis* having over 5% cinnamic acid by weight, dissolved in ethanol; voucher #H2018050305). Borneolum Syntheticum (the synthetic crystal containing mainly borneol not less than 55% by weight, dissolved in ethanol; voucher #180602), and Bufonis Venenum (the dried secretion of *Bufo bufo* gargarizans or *Bufo melanostitus*; voucher #171102). The herbal materials, as well as SBP extracts, were provided by Shanghai Hutchison Pharmaceuticals Company (Shanghai, China). The batch number of SBP was 181006, and the preparation of SBP was performed following Chinese Pharmacopeia 2015. The same batches of SBP used for experiments were stored in Shanghai Hutchison Pharmaceuticals Ltd. The chemical composition of SBP was analyzed by HPLC previously (Xu et al., 2019; Yang et al., 2019). The herbal materials were authenticated morphologically and chemically with referring to Chinese Pharmacopeia 2015. The voucher species were stored at Center for Chinese Medicine at Hong Kong University of Science and Technology (voucher #181202). Dulbecco’s modified Eagle’s medium (DMEM) and fetal bovine serum (FBS) were from Invitrogen (Carlsbad, CA). Dimethyl sulfoxide (DMSO) and donepezil were from Sigma (St. Louis, MO). Purified synthetic Aβ_1–42_ was from GL Biochem (Shanghai, China). The following antibodies Aβ, Bcl-2, Bax, and GAPDH were purchased from Abcam (Danvers, MA). The commercially available sandwich enzyme-linked immunosorbent assay (ELISA) kits of IL-6 and TNF-α were bought from cell signaling technology (Danvers, MA).

The preparation of water (SBP_water_) or 95% ethanol (SBP_EtOH_) extract of SBP was fully described previously (Xu et al., 2019). In brief, 20 g powders of SBP, Bovis Calculus Artifactus, Moschus, Bufonis Venenum, and Cinnamomi Cortex were sequentially sonicated twice in water or 95% ethanol at a proportion of 1:8 (w/v; 160 ml) and 1:6 (w/v; 120 ml) for 30 min each time at 37°C. Total extracts were combined. After dried under vacuum, they were stored at −80°C. The extract of SBP_water_ was dissolved in H_2_O; while SBP_EtOH_ extract, extracts of Borneolum Syntheticum (synthetic having >55% bornel) and Ginseng Radix et Rhizoma were solubilized in dimethylsulfoxide (DMSO). Styrax solution was prepared with DMSO at a ratio of 1:100 (v:v). The extraction was in accord to preparative protocol of SBP (Chinese Pharmacopeia 2015). Stock solutions were at 100 mg/ml, stored at −20°C.

### HPLC Fingerprint

One gram of SBP_water_ or SBP_EtOH_ was dissolved in 10 ml EtOH. The extract was filtered, and the supernatant was collected for chemical analysis (Gao et al., 2008; Xu et al., 2019). The analysis was performed with an Agilent HPLC 1200 system (Agilent, Waldbronn, Germany), equipped with a binary pump, a degasser, an auto sampler, a thermostatic column compartment, and a diode array detector (DAD). The samples were analyzed by using a PLATISIL C_18_ column (4.6 mm × 250 mm, 5 μm i.d.). Acetonitrile (A) and 0.03% phosphoric acid solution (B) were used as the mobile phase according to pre-set gradient program: 0–25 min, linear gradient 15–40% (A), 85–60% (B); 25–55 min, linear gradient 40–75% (A), 60–25% (B); 55–65 min, linear gradient 75–100% (A), 25–0% (B); 65–75 min, 100% (A). The column temperature was 25°C; the injection volume was at 10 μl; the flow rate was set at 0.8 ml/min; and the wavelength was 280 nm.

### Cell Culture and Drug Treatment

PC12 cell line (CRL-1721), derived from rat adrenal medulla, was obtained from American Type Culture Collection (Manassas, VA). The cells were cultured in DMEM, supplemented with 6% horse serum and fetal bovine serum, 100 μg/ml streptomycin, and 100 units/ml penicillin in a humidified CO_2_ (7.5%) incubator at 37°C. In drug treatment, PC12 cells were under serum starvation for 3 h in DMEM containing 1% fetal bovine serum and horse serum. After serum starvation, cells were treated with herbal extract for 48 h. Cell viability assay was used to determine a safe concentration range (0–500 μg/ml) of herbal extract, at which the extracts exerted no effect on cell proliferation or death. The water extracts of SBP, Ginseng extract, and Borneolum Syntheticum solution were used at 500 μg/ml; ethanol extracts and Styrax solution were used at 100 μg/m;. Cell viability was determined by MTT assay. In brief, cells were plated in 96-well plate at a density of 10,000 cells/well for 24 h and treated with drugs for 48 h. Then, 10 μl MTT solution, at a concentration of 5 mg/ml, was added into each well, incubated for another 3 h at 37°C. Using a microplate reader (Thermo Fisher Scientific, Waltham, MA), the absorbance was measured at 570 nm (Lam et al., 2016; Lam et al., 2017).

### Preparation of Aβ_1-42_

Preparation of Aβ_1-42_ peptide was performed as previously reported (Yang et al., 2005). Briefly, to obtain monomeric Aβ_1-42_, the lyophilized Aβ_1-42_ was dissolved in 100% 1,1,1,3,3,3-hexafluoro-2-propanal (HFIP) and sonicated in a water bath for 10 min. Then, the Aβ_1-42_ solution was subsequently stored in small aliquots at −80°C. Prior to use, the HFIP-treated monomeric Aβ_1-42_ was immediately dried in the fume hood and dissolved in sterilized distilled water at a stocking concentration of 1 mM.

### ThT Fluorescence Assay

The amounts of Aβ_1-42_ fibrils resulting from aggregation of monomeric Aβ_1-42_ were determined by Thioflavine T (ThT) assay, in which the fluorescence intensity represented the degree of Aβ_1-42_ fibril formation (Yang et al., 2005; Kroth et al., 2012). Aβ_1-42_ was diluted to 10 μM in PBS in brown microtube in the presence or absence of curcumin (30 μM), SBP extracts or other herbal extracts, and then which was incubated at 37°C for 6 days without agitation. Ten μl of Aβ_1-42_ solution was mixed with 90 μl of 5 μM ThT in PBS, and the fluorescence intensity was measured at excitation and emission wavelength of 435 and 488 nm, respectively, with application of 96-well black plate.

### MTT Assay

Cell viability was measured by MTT assay (Li et al., 2007; Choi et al., 2010). In brief, 24 h after cell seeding, the culture medium was replaced with FBS-free medium, and then Aβ_1-42_ samples (15 μM) (co-incubated with or without curcumin, SBP extracts and herbal extracts at 37°C for 6 days) were added to the cultures. Forty-eight hours after incubation, 10 μl MTT, at a concentration of 5 mg/ml in PBS, was added into each well, and the cells were incubated for another 4 h. The formed formazan crystals were dissolved in DMSO, and the absorbance was determined using a microplate reader with wavelength at 570 nm.

### Animal Treatment

The APP/PS1 transgenic mice and B6C3 wild type mice (all male) were obtained from Hangzhou Hibio company (Hangzhou, China). The mice were housed at two to three animals per cage under a 12 h light/dark cycle, and they were fed with standard food and water. The experimental protocols were approved by Animal Ethics Committee (Permit Number: HB1808021) and under the guidelines of “Principles of Laboratory Animal Care” (NIH publication No. 80-23, revised 1996) and Institutional Animal Care and Use Committees protocol (HBFM3.68-2015.). Five-month-old APP/PS1 transgenic mice were randomly divided into five groups: the model group, donepezil (2 mg/kg/day), SBP_EtOH_ (95% ethanol extract of SBP) low-dosage group (22.5 mg/kg/day), SBP_EtOH_ high-dosage group (45 mg/kg/day). Age-matched male wild-type (WT) B6C3 mice were used as the control group (*n* = 6). In the APP/PS1 mice model group and WT mice group, the mice were injected with corresponding volume of saline. Donepezil, dissolved in saline, and the SBP solution (2% DMSO, 6% cremophor EL, 92% NaCl) were administered intra-gastrically once per day. Drug treatment lasted for 60 days.

### Morris Water Maze Test

Morris water maze test was performed, as described previously (Xian et al., 2014). The experimental apparatus composed of a blank circular water tank, and the diameter and height of the water tank was 100 and 35 cm, respectively. The depth of water (23 ± 1°C) in the tank was ~15.5 cm, and by adding ink, it was rendered opaque. The maze was divided into four equal quadrants by four poles along the perimeter of pool. A platform, of which the diameter was 4.5 cm and the height was 14.5 cm, was submerged about 1 cm below the water surface and set at the middle of one quadrant. The pool was placed in a test room, and there were various visual cues needed, e.g. lamps, pictures, etc. Twenty-four hours prior to the spatial training, the mice suffered four pre-training sessions as followed: the mice was placed on the platform for 20 s, given a 30 s free swim, and then assisted to the platform, where it was permitted to have a rest for another 20 s. Spatial training to find the platform hidden in the water maze was performed consecutively for 4 days. Each day, the mice was put in water in face of the pool wall at one of three randomized starting positions, of which they did not contain the platform. The mice were allowed 60 s to catch the platform and given 20 s to stay on it. Animals failing to find the platform in a period of 60 s were manually guided to the platform and given 20 s to stay. The escape latency for finding the submerged platform was regarded as 60 s. Four trials were performed for each mouse per day, and the inter-trial interval lasted for 60 s. The escape latency to the submerged platform of each trial was recorded, and the average value of 4 trials was analyzed. Twenty-four hours after the final time of spatial training, the probe test was performed by removing the platform and giving each mouse to swim freely for 60 s. The time that each mouse spent swimming in the target quadrant, where the platform was once hidden previously, and the number of times (frequency) that it crossed over the platform site were recorded.

### Histochemistry

Mice were anesthetized with chloral hydrate (100 mg/kg) 24 h after performing the behavioral test, and then the mice were perfused with ice-cold saline. The brains were dissected into two parts: one was frozen at −80°C for protein analysis and western blotting; the other was being fixation in 4% paraformaldehyde (pH 7.4) at 4°C overnight for immunohistochemistry. In sectioning, the fixed brains were successively dehydrated in ethanol, embedded in paraffin, and cut into 4 μm-thick sections. The sections were treated with 0.01 M sodium citrate for 15 min at 95°C and incubated with 3% H_2_O_2_ for 8 min. The sections were then reacted with primary rabbit antibody against Aβ (rabbit polyclonal, 1:100, Abcam) at 4°C, followed by interaction with horseradish peroxidase-conjugated goat anti-rabbit secondary antibody (1:200, Abcam), and finally visualized with diaminobenzidine tetrahydrochloride. The expression of Aβ was analyzed using Image-Pro Plus software. Five sections were randomly selected from each mouse.

### Morphological Analysis

After fixation, the brain tissues were successively dehydrated in 80, 90, 95, and 100% ethanol. The tissues were embedded in paraffin for 5 h. The paraformaldehyde/paraffin-embedded samples were then sectioned to 4-μM thickness and mounted on microscope slides. The hematoxylin stain was used to dip the sections, and after that, slides were washed with water until the sections became blue. Next, the sections were immersed in eosin staining solution for 3–5 min and then washed with water again, until there was no floating color on the slides. After dehydration, the slides were fixed with neutral glue. Neuronal counts, located in the hippocampus, were performed under a microscope. The nucleus was blue, the cytoplasm was pink, and the red blood cells were bright red. Counts were performed in five to six adjacent fields, and all data are represented as number of neurons under each observation field.

### Determination of Biomarkers

For determination of levels of NO, NOS, and AChE, 0.1 g of brain tissue was homogenized using 0.9 ml of pre-cold saline. After reacted on ice for 15 min, the homogenate was centrifuged at 2,500 rpm/min for 10 min at 4°C, and the supernatants were used to measure the levels of NO, NOS, and AChE with application of corresponding kits according to the manufacturer’s instructions. To measure the level of NO, 300 μl homogenate was reacted with reagents in a NO kit (A013-2-1, Jiancheng Bio, Nanjing, China). After standing for 10 min, the mixture was centrifuged at 3,500 rpm/min for 15 min, and 150 μl supernatant was used to determine NO level with wavelength set at 550 nm. The wavelength to determine the level of NOS was set at 530 nm (A014-2, Jiancheng Bio), and the level of AChE was determined at the wavelength of 412 nm (A024-1-1, Jiancheng Bio). For the measurement of cytokines in mice, the blood was collected from the removal of eyeballs under anesthesia. After standing for 1 h at 37°C, the blood was centrifuged at 3,500 rpm/min for 10 min, and the serum was obtained. The serum was further used to determine the levels of IL-6 and TNF-α by using commercially available sandwich enzyme-linked immunosorbent assay (ELISA) kits (Invitrogen, Carlsbad, CA), following the manufacturer’s manual.

### RT-PCR Analysis

Total RNA of the hippocampus and cortex was extracted using RNazol following the manufacturer’s instructions (Takara, Dalian, China): the concentration of RNA was determined spectrophotometrically at 260 nm. The quality of RNA was further assessed by the ratio of absorbance at 260 and 280 nm. The values of A260/A280 from 1.9 to 2.1 were considered as reasonable. Total RNA (1.5 μg) was applied to synthesize cDNA using HiScript-II Q RT SuperMix for qPCR kit (Vazyme Biotech, Nanjing, China) following the manufacturer’s manual, and RT-PCR was performed using reagents and protocols from ChamQ SYBR Color qPCR Master Mix kit (Vazyme Biotech). The sequences of primers were shown: 5’-GCC TCC TCT CCT ACT TCG G-3′ (sense primer, S) and 5′-TCA GCC CAT CTT CTT CCA G-3′ (antisense primer, AS) for Bax; 5′- AAA CCC TCC ATC CTG TCC-3′ (S) and 5′-TCC TAA ACC CTG CTT CCC-3′ (AS) for murine Bcl-2; 5′-GAA GCA GGC ATC TGA GGG-3′ (S) and 5′-AAG GTG GAA GAG TGG GAG TT-3′(AS) for murine GAPDH. The relative expressions of Bax and Bcl-2 mRNA were normalized to the amount of GAPDH in the same cDNA following the relative quantification method (2^-ΔΔCT^ method).

### Western Blotting

Protein extracts were extracted from frozen brain tissues using Nuclear and Cytosolic Protein Extraction kit (Chemicon, Temecula, CA), and protein concentrations were measured using the BCA protein assay kit based on protocol provided by the manufacturer. The protein samples were electrophoresed by sodium dodecyl sulfate-polyacrylamide gel electrophoresis (SDS-PAGE) for 3 h at 80 V, and then separated proteins were transferred onto polyvinylidene fluoride (PVDF) membranes. Next, the membranes were blocked with 5% (w/v) non-fat milk in TBS-T (Tris-buffer saline containing 0.1% Tween-20) at room temperature for 1 h. After blocking, the membranes were incubated at 4°C overnight with appropriate amounts of primary antibodies against Aβ, Bax, Bcl-2 (1:1,000, Abcam, Danvers, MA), and GAPDH (1:5,000, Abcam). Then, the membrane was rinsed with TBS-T three times, 5 mins per each time, and probed with horseradish peroxidase conjugated secondary antibody at room temperature for 2 h. To prove equal loading of samples, the membranes were incubated with monoclonal antibody GAPDH. Finally, the protein bands were visualized using the ECL Western blotting detection reagents (Amersham Biosciences, Buckinghamshire). The intensity of each band was analyzed with application of Image J software (NIH Image, Bethesda, MD).

### Statistics Analysis

Statistical tests were performed using one-way analysis of variance (ANOVA); differences from basal or control values were classified as * *p* < 0.05; ** *p* < 0.01; *** *p* < 0.001.

## Results

### SBP Inhibits Aβ_1-42_ Fibrils and Protects Cell Damage

Previous records suggested that SBP_water_ and SBP_EtOH_ extracts were rather effective in triggering differentiation of cultured PC12 cells (Xu et al., 2019), and thus both of the extracts were subjected for further analyzed. The HPLC chromatogram of SBP_water_ and SBP_EtOH_ and 12 major peaks were identified (**Supplementary Figure 1**). The chemical information of non-volatile constituents in SBP was shown characteristically in the chromatogram, and the authenticated 12 peaks were precisely confirmed by comparison with known chemical standards, as reported previously (**Supplementary Figure 1** **;** Xu et al., 2019). The demonstrated HPLC chromatogram, as well as its chemical composition, could be used as fingerprint of SBP and other herbal extracts for quality control purpose, as to ensure the repeatability of biochemical experiments.

**Figure 1:**
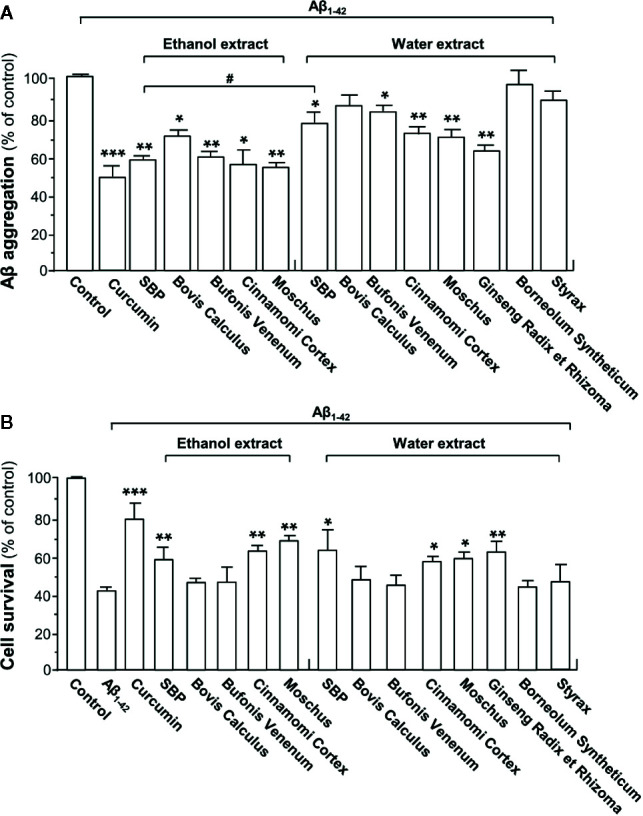
SBP suppresses Aβ fibril formation and attenuated Aβ-induced toxicity in PC12 cells. **(A)** HFIP-reconstituted Aβ monomers (10 μM) were incubated at 37°C for 6 days with or without drugs (i.e. curcumin, SBP_EtOH_ or SBP_water_, and other seven individual herbal materials). SBP_EtOH_, ethanol extracts of various herbal materials, and Styrax solution were used at 100 μg/ml; and SBP_water_, water extracts of various herbal materials, solutions of Ginseng Radix et Rhizoma extract and Borneolum Syntheticum were used at 500 μg/ml. Curcumin at 30 μM served as a positive control. The extent of Aβ aggregation was determined using ThT fluorescence assay. Data are expressed as Mean ± SEM of the percentage of control (no drug treatment), where *n* = 4; *p* < 0.05 (*); *p* < 0.01 (**); *p* < 0.001 (***) *vs* control group. *p* < 0.05 (^#^) *vs* SBP_EtOH_ group. **(B)** Inhibition of cytotoxicity in PC12 cells by drug-modified Aβ_1-42_ aggregates. Aβ_1-42_ (15 μM) was incubated at 37°C for 6 days in the presence or absence of drugs (i.e. curcumin, SBP_EtOH_ or SBP_water_, and other seven individual herbal materials), and then treated to PC12 cells for 48 h. The doses were in (A). MTT assay was performed to determine the cell viability. Data are expressed as Mean ± SEM of the percentage of control (untreated culture), where *n* = 4; *p* < 0.05 (*); *p* < 0.01 (**); *p* < 0.001 (***) *vs* Aβ_1-42_-treated group.

To evaluate the ability of each herbal extract (i.e. curcumin, SBP_EtOH,_ or SBP_water_ and other seven individual herbal materials) in suppressing formation of Aβ_1-42_ fibrils, the monomeric Aβ_1-42_ was incubated with curcumin (a positive control), SBP_water_ or SBP_EtOH_ or other seven individual herbal extracts at 37°C for 6 consecutive days, as such allowing Aβ_1-42_ to age. As shown in **Figure 1A**, neither water extract of Bovis Calculus nor 95% ethanol extracts of Borneolum Syntheticum and Styrax showed effect on formation of Aβ_1-42_ fibrils. In contrast, SBP_water_, SBP_EtOH_, Bufonis Venenum extract, Cortex Cinnamomi extract, Moschus and Ginseng Radix et Rhizoma extract, and ethanol extract of Bovis Calculus, significantly suppressed Aβ_1-42_ fibril formation with decreasing level of ThT fluorescence. The ethanol extracts of SBP, Bovis Calculus, Bufonis Venenum, Cortex Cinnamomi, and Moschus demonstrated strong capability in inhibiting formation of Aβ_1-42_ fibrils than their water extracts **(** **Figure 1A** **)**. The ethanol extract of SBP and Moschus showed better efficacy in suppressing Aβ_1-42_ fibril formation. Curcumin, as a positive control, decreased the fluorescence intensity significantly, consistent with previous studies (Ono et al., 2004; Yang et al., 2005).

To determine toxicity of Aβ_1-42_ aggregate being aged with drugs, Aβ_1-42_ was incubated with curcumin, SBP_EtOH_ or SBP_water_ or other seven individual herbal extracts at 37°C for 6 days, and then which were used to treat cultured PC12 cells for 48 h. The treatment with Aβ_1-42_ fibrils significantly decreased cell viability by ~60% **(** **Figure 1B**). In comparison to Aβ_1-42_ fibril, Aβ_1-42_ having incubation with SBP_water_, SBP_EtOH_, extracts of Cortex Cinnamomi, Moschus, and Ginseng Radix et Rhizoma showed less toxicity to cultured PC12 cells, similar to that of curcumin. As compared to the water extract, the ethanol extract of Bufonis Venenum demonstrated significant effect in relieving Aβ_1-42_-induced cell toxicity **(** **Figure 1B** **)**. In contrast, the water extracts of Bovis Calculus, Bufonis Venenum, Borneolum Syntheticum, and Styrax were unable to protect the Aβ_1-42_-induced cell toxicity **(** **Figure 1B** **)**. The SBP-protected cell death against Aβ fibrils probably due to the ability of herbal extract in reducing the fibrilization. In terms of dose and efficacy, SBP_EtOH_ extract showed better response as compared to that of SBP_water_ extract, and therefore SBP_EtOH_ was chosen for animal study.

### SBP Rescues Behavioral Impairment

**Figure 2:**
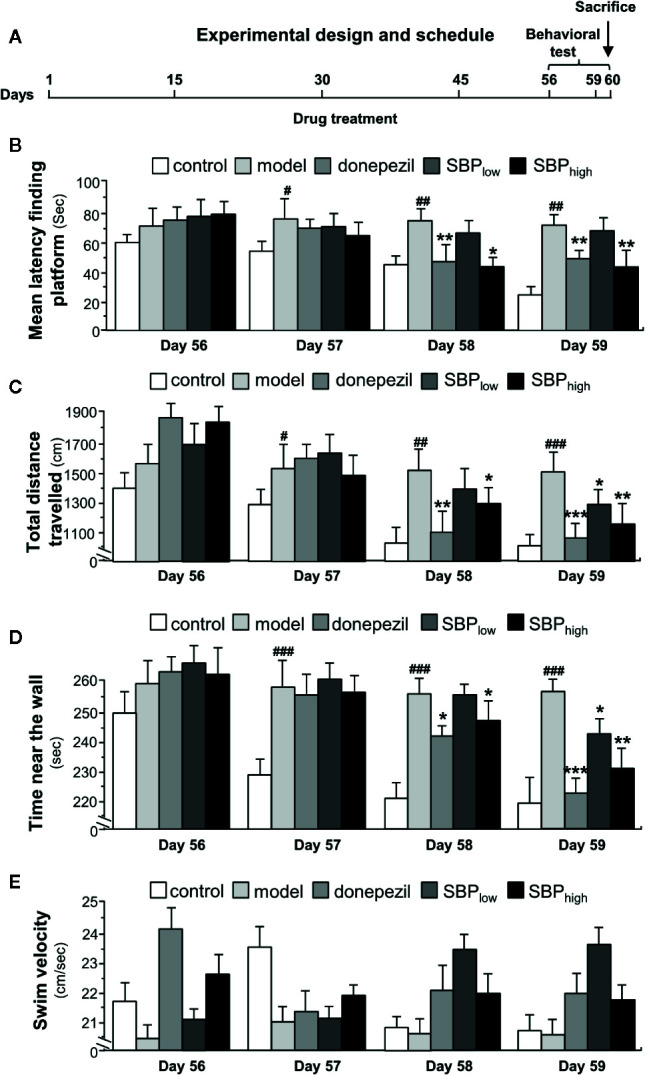
The schedule of animal experiment. **(A)** The APP/PS1 transgenic mice and B6C3 wild type mice (all male at 5-month-old) were fed with standard food and water. The transgenic mice were randomly divided into five groups, i.e. model group, donepezil (2 mg/kg/day), SBP_EtOH_ (95% ethanol extract of SBP) low-dosage group (22.5 mg/kg/day), SBP_EtOH_ high-dosage group (45 mg/kg/day). Age-matched male wild-type (WT) B6C3 mice were used as control group (*n* = 6). In APP/PS1 mice model group and wild type mice group, the mice were injected with the corresponding volume of saline. Donepezil, dissolved in saline, and the SBP extract (2% DMSO, 6% cremophor EL, 92% NaCl) were administered intra-gastrically once per day starting on Day 1. Drug treatment lasted for 60 days. Behavioral test was started on Day 56. **(B)** The treatment of mice with herbal extracts were according to the protocol in Figure 2A. The latency to find a hidden platform during 4 consecutive days of training was determined in Morris water maze. **(C)** The total distance of swimming-tracking path during 4 consecutive days of training was determined. **(D)** The time near the wall spent in target quadrant (southeast, where the platform was placed during the training phase) during 4 consecutive days of training was determined. **(E)** The swim velocity during 4 consecutive days of training was determined. Values were expressed as mean ± SEM, where *n* = 6; *p* < 0.05 (^#^); *p* < 0.01 (^##^); *p* < 0.001 (^###^) *vs* control group. *p < 0.05; **p < 0.01; ***p < 0.001 *vs* model group.

The experimental protocol for treatment with SBP_EtOH_ extract in mice was shown in **Figure 2A**. The behavioral test was performed in 56 days after the daily intake of herbal extract/drug. Mice were sacrificed at day 60 for biochemical analysis. Morris water maze was employed to reveal the dysfunction of spatial learning and memory. The shortened escape latency was found in the control mice during successive days of training; but which was not found in APP/PS1 transgenic mice, suggesting the establishment of behavioral model **(** **Figure 2B** **)**. The performance of drug-treated mice was improved through the training (Day 57–Day 59). The escape latency in APP/PS1 transgenic mice was higher than that of control group (non-transgenic; wild type), suggesting the transgenic mice took relatively longer time to find the hidden platform. As compared to model group, the treatment of SBP at high dose in APP/PS1 transgenic mice significantly shortened the mean latency to find the platform by ~50% from the Day 58 onward **(** **Figure 2B** **)**. In addition, treatment of donepezil significantly reduced the mean latency after Day 58. The low dose of SBP however did not decrease the mean latency, significantly. In addition, total distance traveled, swim velocity, and time near wall during the training period were measured (**Figures 2C–E** **)**. As demonstrated, the treatment of donepezil significantly reduced the total distance of the swimming-tracking path **(** **Figure 2C** **),** as well as the time near the wall spent in target quadrant **(** **Figure 2D** **)**. In comparison with model group, the treatment of SBP at low and high doses in APP/PS1 transgenic mice obviously decreased the total distance of the swimming-tracking path **(** **Figure 2C** **)** and the time near the wall spent in target quadrant after Day 58 **(** **Figure 2D** **)**. Donepezil treatment significantly reduced the total distance and the time near the wall after Day 57. However, there was no significant difference between SBP-treated groups and model group in terms of swim velocity **(** **Figure 2E** **)**.

**Figure 3:**
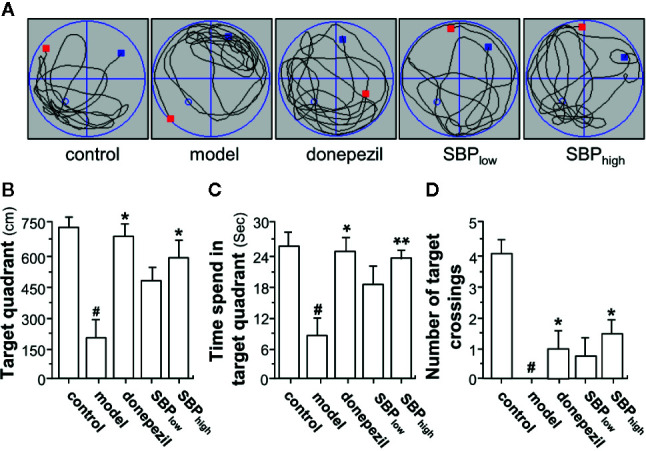
SBP rescues memory deficits in APP/PS1 transgenic mice. **(A)** The treatment of mice with herbal extracts were according to the protocol in **Figure 2**. The typical swimming-tracking path on the fifth probe trial day was determined. The red square represents the place where the animal is being placed at the beginning of water maze test. The blue square represents the place where the animal is stopped at the end of water maze test. The blue circle represents the place where the platform is placed before water maze test. **(B)** the distance in target quadrant (southeast, where the platform had been placed during the training phase) in the probe trial (swimming 60 s without platform) was determined. **(C)** the time spent in the target quadrant was determined. **(D)** the number of target crossings in the target quadrant was determined. Values were expressed as mean ± SEM, where *n* = 6; *p* < 0.05 (^#^) *vs* control group. *p < 0.05; **p < 0.01 *vs* model group.

In the probe test, the distance, the time spent in target quadrant and the number of target crossings of mouse in Morris water maze were significantly reduced in APP/PS1 transgenic mice, as compared to the wild type mice **(** **Figures 3A–D** **)**. The mice having treatment of SBP, at low and high doses, induced an increase in the distance, the time spent in the target quadrant, and the number of target crossings, as compared to the model group. In addition, the mice having SBP treatment at high dose demonstrated a significant difference in the probe test **(** **Figures 3A–D** **)**. Donepezil, a positive control, significantly increased the distance and the time spent in target quadrant, as well as the number of target crossings of the transgenic mice. The high dose of SBP demonstrated better effect in increasing the number of target crossings than that of donepezil-treated group.

### SBP Rescues Pathological Changes in APP/PS1 Mice

**Figure 4:**
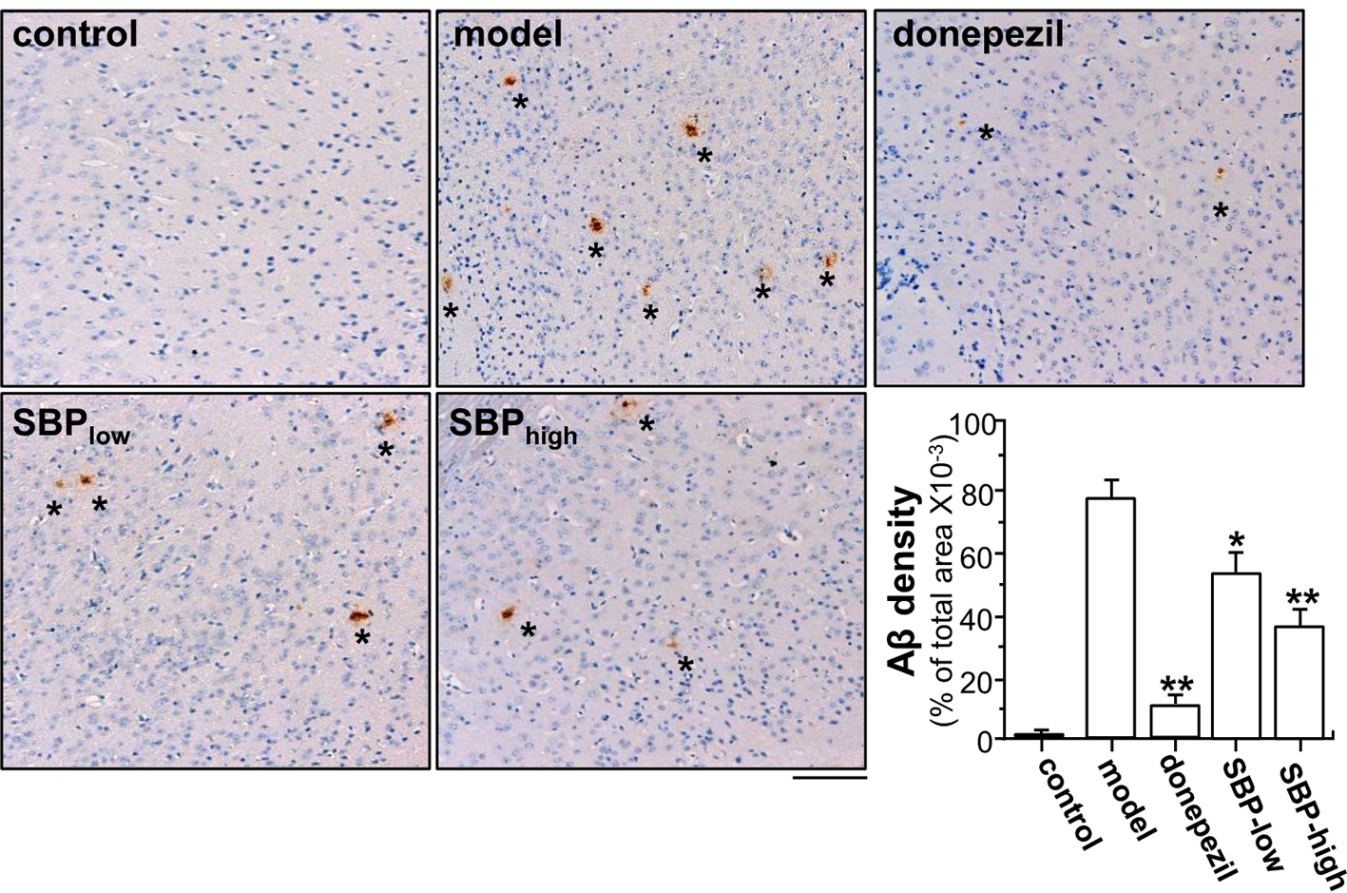
SBP reduces Aβ expression in brain tissue. Brain tissues from drug-treated mice were collected from mice as described in **Figure 2**. Brain tissues were immuno-stained with anti-Aβ antibody. The cerebral cortex was selected for analysis, as indicated **Supplementary Figure 2**. Taupe area (star) represented the antibody staining. ImageJ software was used to do the quantification of Aβ plaque area. The control group was untreated wild type mice. Data are in Mean ± SEM of the Aβ density in terms of percentage of total area per view, where *n* = 6; *p* < 0.05 (*); *p* < 0.01 (**) *vs* model group. Bar = 100 μm.

After behavioral analysis, the mouse was scarified, and the cross-section of brain was subjected to immunohistochemical studies. Two specific areas of the section, cerebral cortex and hippocampus, were chosen for analysis (**Supplementary Figure 2**). Immunohistochemistry revealed the robust identification of Aβ in cerebral cortex of the transgenic mice **(** **Figure 4** **)**. APP/PS1 mice showed high level of Aβ plaque in cerebral cortex, as compared to that of control group. Donepezil decreased Aβ plaque by ~90% in APP/PS1 mice, as compared to model group **(** **Figure 4** **)**. In parallel, the treatments of SBP, at low and high doses, inhibited formation of Aβ plaque by ~40 and ~60%, respectively, in the mouse brain: both doses were significant, as compared to control **(** **Figure 4** **)**.

**Figure 5:**
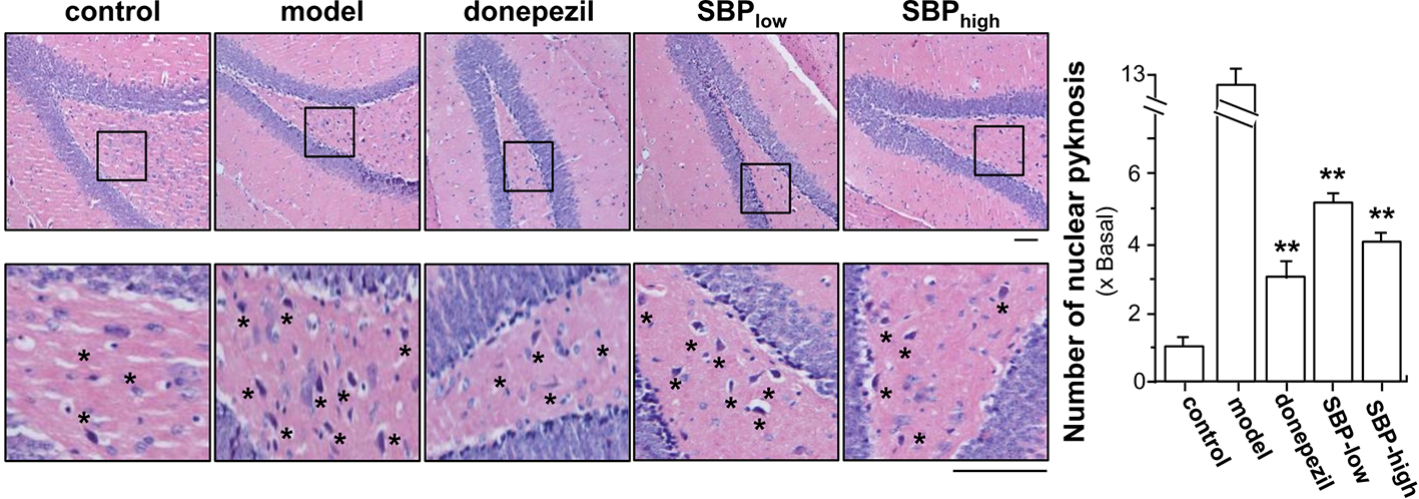
SBP protects neurons from nuclear pyknosis in APP/PS1 transgenic mice. Brain tissues from drug-treated mice were collected from mice as described in **Figure 2**. The hippocampus was selected for analysis, as indicated **Supplementary Figure 2**. Brain tissues were collected and cut into sections at 4 μM thick. The sections were stained with hematoxylin and eosin stains. Typical pictures focusing the hippocampus were shown, in which the nucleus was blue, the cytoplasm was pink, and the red blood cells were bright red (left panel). The number of neurons having nuclear pyknosis (denoted as star for some but not all, for clarity) was determined under light microscope using Image proplus program (right panel). Results are expressed as the fold of change as compared to control (X Basal; untreated wild type mice), where the control was set as 1, Mean ± SEM, where *n* = 6; *p* < 0.01 (**) *vs* model group. Bar = 50 μm.

In addition, the effect of SBP on nuclear pyknosis of neurons in hippocampus of the transgenic mice was analyzed. The number of neurons suffering from nuclear pyknosis in APP/PS1 transgenic mice increased by ~13 folds, as compared to the mice in control group (**Figure 5**). SBP administration, at low and high doses, significantly decreased the number of neurons suffering from nuclear pyknosis to 4–5 folds of control mice, i.e. a reduction of over 2 folds from model group (**Figure 5**). Comparing to mice in model group, donepezil could attenuate the neurons experiencing nuclear pyknosis to ~3 folds of control.

**Figure 6:**
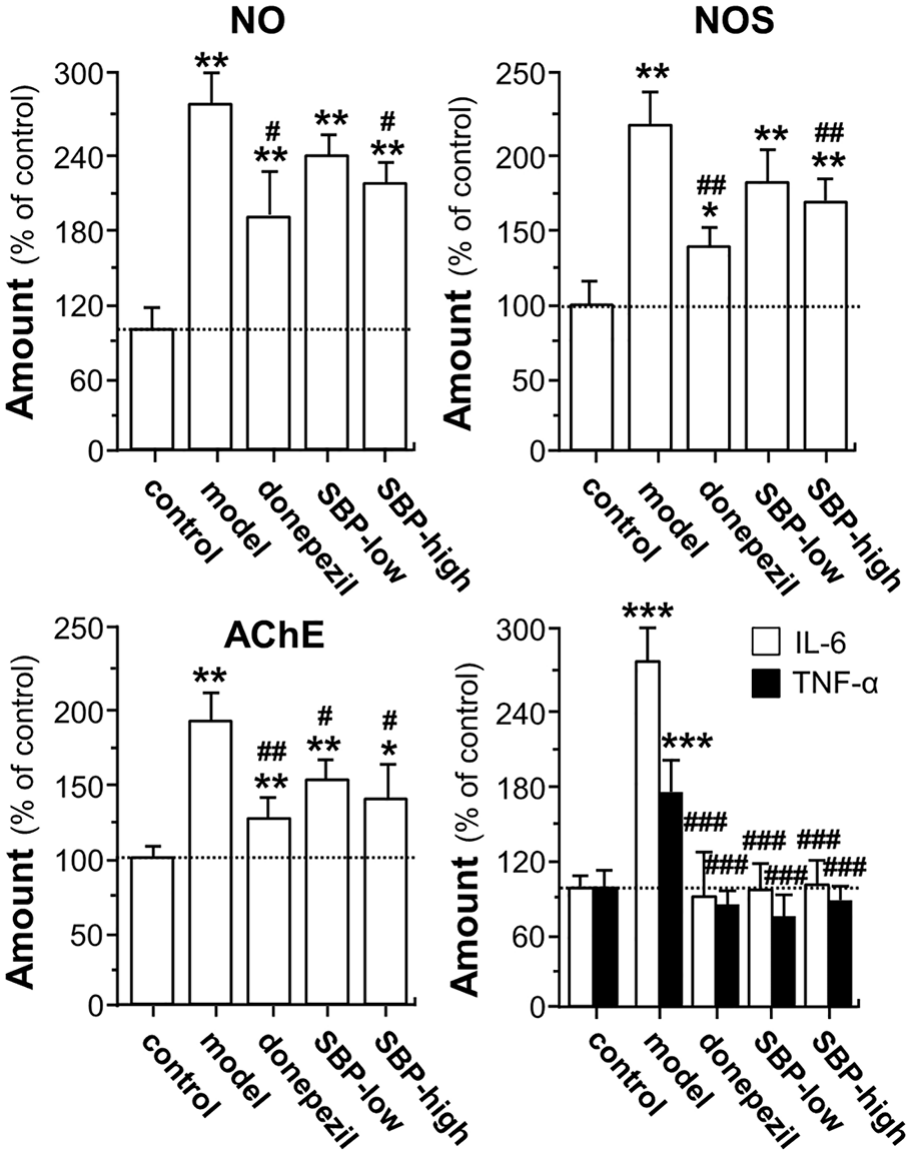
SBP promotes expressions of NO, NOS, AChE, and cytokine in APP/PS1 transgenic mice. Treatment of mice was as described in **Figure 2**. For determination of levels of NO, NOS, and AChE, 0.1 g of brain tissue was homogenized using 0.9 ml of pre-cold saline. After reacted on ice for 15 min, the homogenate was centrifuged at 2,500 rpm/min for 10 min at 4°C, and the supernatants were used to measure the levels of NO, NOS, and AChE with application of corresponding kits according to the manufacturer’s instructions. Blood was collected for the measurement of the amounts of IL-6 and TNF-α. The blood was centrifuged at 3,500 rpm/min for 10 min, and the serum was obtained by discarding the precipitant. The levels of IL-6 and TNF-α in the serum was further determined using commercially available ELISA kits. Data are expressed as Mean ± SEM of the percentage of change as compared with control (untreated wild type mice), where *n* = 6; *p* < 0.05 (*); *p* < 0.01 (**); *p* < 0.001 (***) *vs* control group. *p* < 0.05 (#); *p* < 0.01 (##); *p* < 0.001 (###) *vs* model group.

The suppressive effects of SBP on protein levels of indicative biomarkers of AD mouse brain, i.e. NO, NOS, and AChE, were investigated. As shown in **Figure 6**, when compared to control group, protein levels of NO, NOS, and AChE in the brain of APP/PS1 transgenic mice were significantly elevated, at least over 2 folds. On the other hand, the treatment of SBP significantly reduced the expressions of NO, NOS, and AChE, as compared with the model group, i.e. un-treated APP/PS1 mice. The treatment of SBP at high dose decreased the levels of NO, NOS, and AChE by ~70, ~50, and 40%, respectively. In addition, the treatment with donepezil reduced the protein levels of NO, NOS, and AChE **(** **Figure 6** **)**. Moreover, we further examined the expressions of IL-6 and TNF-α in the mouse serum by performing an ELISA assay. In serum of APP/PS1 mice, the levels of IL-6 and TNF-α were increased at least by 2 folds (**Figure 6**). In addition, the inflammatory cytokine was determined in the SBP-treated mice. The treatment of SBP, at both low and high doses, was able to suppress the up-regulated cytokines in APP/PS1 mice (**Figure 6**). Similar to that of SBP, donepezil was able to decrease the expressions of IL-6 and TNF-α.

### SBP Exerts Neuroprotective Effects *via* Anti-Apoptotic Pathway

**Figure 7:**
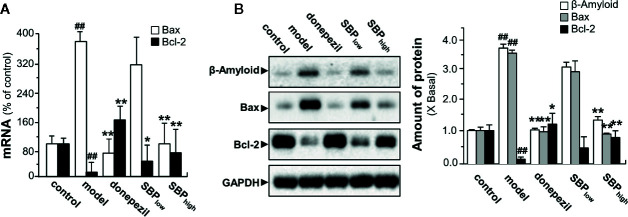
SBP promotes neuroprotective effects *via* anti-apoptotic pathway. Treatment of mice was as described in **Figure 2**. **(A)** Effects of SBP on the mRNA expressions of Bax and Bcl-2 in the brains in APP/PS1 transgenic mice. Data are expressed as Mean ± SEM of the percentage of change (up or down regulated) as compared with control (untreated wild type mice), where *n* = 6; *p* < 0.01 (^##^) *vs* control group; *p* < 0.05 (*); *p* < 0.01 (**) model group. **(B)** Expression of β-Amyloid (~87 kDa), Bax (~21 kDa), and Bcl-2 (~26 kDa) in brain tissues of drug-treated mice (left panel). Expression of GAPDH (~36 kDa) served as a control. Quantitation of protein expression is shown (right panel). Results are expressed as the fold of change as compared to control (X Basal; untreated wild type mice), where the control was set as 1, Mean ± SEM, where *p* < 0.01 (^##^) *vs* control group; *n* = 4; *p* < 0.05 (*); *p* < 0.01 (**) *vs* model group.

To determine the possible anti-apoptotic signaling of SBP in neuroprotection, RT-PCR and western blotting assay were performed to determine expression of apoptotic genes. In APP/PS1 mice, the mRNA expression of Bax was increased by ~4 folds, as compared to wild type mice; while the mRNA level of Bcl-2 was decreased by ~90% (**Figure 7A**). In SBP-treated APP/PS1 mice, the expression of Bax was significantly reduced under a high dose of SBP intake, almost back to control level (**Figure 7A**). On the other hand, SBP treatment increased the mRNA level of Bcl-2 at both doses of SBP. As expected, the treatment with donepezil significantly altered the mRNA expressions of Bax and Bcl-2. The protein expressions of Bax and Bcl-2 demonstrated similar trends in western blotting analysis. In the brain of APP/PS1 mice, the expression levels of Aβ and Bax were increased markedly over 3 folds (**Figure 7B**). The treatment of SBP (high dose) in the transgenic mice significantly decreased the protein levels of Aβ and Bax almost back to the control. In parallel, the reduced Bcl-2 protein in APP/PS1 mice was restored by SBP (high dose) almost back to control level **(** **Figure 7B** **)**. Similarly, donepezil significantly decreased the ratio of Bax/Bcl-2. These results suggested the function of SBP in protecting neuron could be mediated by an anti-apoptotic pathway.

## Discussion

Very limited effective drugs are available for treatment of AD, and the current used single-targeted anti-AD drugs might only offer symptom relieving effects. The major reason could be that AD is caused by multiple factors; thus, safe and effective multi-functional anti-AD drug should be urgently needed (Scheltens et al., 2016). Amongst those multi-components of Chinse herbal medicine, SBP is a well-known herbal mixture being sold in the current herbal market for years. This herbal mixture is mainly used in treating cardiovascular diseases (Yan et al., 2006; Lu et al., 2018). Here, we are proposing another possible application of SBP in treating neurodegenerative diseases. In cultured neuronal cells, we have reported that SBP could induce neuro-differentiation, as well as cell protection, *via* activation of cAMP-PKA pathway (Xu et al., 2019). Our hypothesis of SBP as a new and promising anti-AD drug is supported by different lines of evidence. First, SBP exhibited neurite-outgrowth promoting activity in cultured neuronal cells, as reported previously (Xu et al., 2019). Second, SBP reduced Aβ aggregation, as well as the Aβ-induced neurotoxicity cultured cells. Third, SBP reduced amyloid deposition, as well as reduced expressions of NO, NOS, AChE in the brain. Several lines of evidence support the involvement of neuroinflammation playing a substantial role in development of neuropathological changes in AD patients (Rogers et al., 1988; Griffin et al., 1989). Reports have suggested that the anti-inflammatory treatments for inflammation-related diseases, such as rheumatoid arthritis, could show protective effects against AD development, i.e. up to 50% reduction in the risk of developing AD among those patients taking non-steroidal anti-inflammatory drugs (Rich et al., 1995). Having this notion, the anti-inflammatory effects of SBP could be accounted for anti-AD function in ours model, at least part of that. As demonstrated here, SBP suppressed production of inflammatory factors (IL-6 and TNFα) in serum of APP/PS1 transgenic mice. Last, SBP improved cognitive impairment of APP/PS1 transgenic mice. Under this scenario, SBP could be an excellent choice for AD treatment, or even as preventing agent. For a good reason, SBP has been on herbal market for years, and millions of people have taken this herbal mixture during the years, which therefore guarantees its safety.

In the past decade, the over production and accumulation of Aβ in the brain have been hypothesized to play a crucial role in pathological cascades of AD. The *in vitro* data have indicated that SBP prevented Aβ-induced neurotoxicity by inhibiting the fibrilization process. In recent target for drug screening, drug having ability to reduce deposition and neurotoxicity of Aβ in brain could be potential anti-AD agents; however, few of them have achieved the final success (Wharton et al., 2012; Egan et al., 2019). One of the obstacles is that the drug candidates have shown promising positive results in *in vitro* model but not in *in vivo* model. The animal model of AD is not a straightforward issue, and several such disease models have been proposed. APP/PS1 transgenic mouse is one the most popular animal models to mimic the cognitive impairment of AD patients. APP/PS1 transgenic mouse, bearing double mutations on the genes of APP and PSEN1, develops amyloid plaque in neocortex at ~6 weeks of age and amyloid deposits in hippocampus at ~5 months (Lok et al., 2013). Moreover, APP/PS1 transgenic mouse exhibits cognitive impairments, and which can be examined by behavioral assays. SBP, a possible anti-AD candidate, processes activities in both *in vitro* and *in vivo* models, supporting its further exploration.

The development of AD is believed to be caused by multiple factors, and single-targeted drug is only offering limited symptom-relieve effect but not restore or delay the pathological progress. Having multiple-targeted therapeutic strategy therefore could provide synergy in treating the complex neurodegenerative disease. FDA has not approved new anti-AD drug since 2001, except a combination of AChE inhibitor and NMDA receptor antagonist in 2014. Many anti-AD drugs targeting various proteins are failed during the clinical trial because of its efficacy. Thus, a new direction to develop AD drug is in urgent need, and therefore the drug candidate having multi-functional target is a common approach. These candidates with disease-modifying potential may delay or reverse the pathological progress of AD. In accordance to this notion, TCM is effective and safe for treatment of many diseases (Gao et al., 2008; Jiang et al., 2013). Indeed, SBP is a herbal mixture, which is having multiple functions, including promotion of neurogenesis and neuroprotection, through regulating different therapeutic targets. The results of biochemical assays in APP/PS1 transgenic mice have supported our hypothesis. The elevated expressions of Aβ, NO, and NOS in the brain of APP/PS1 transgenic mice were effectively reduced by SBP. In addition, AChE, an enzyme target for several anti-AD drugs, was reduced by SBP, suggesting the cholinergic transmission might be enhanced. To account the efficacy of SBP in AD mice, the individual ingredients of SBP have been proposed to play role in the brain functions. SBP composes of eight herbal extracts, many of them are being applied in clinics for brain protection. For example, Moschus and Borneolum Synthcticum, known as a drug-pair in TCM, are known to treat cerebral stroke (Xiang et al., 2012). Moreover, the components from ginseng have been shown to have neuroprotective effects by suppressing the ROS formation and mitochondrial bioenergetics (Huang et al., 2019). Ginsenoside Re exerted anti-inflammatory effects by suppressing the formation of nitric oxide and attenuating the NF-κB signaling in the LPS-induced microglial cell (Wu et al., 2007). In parallel, ginsenoside Rg1 decreased the level of Aβ_1-42_ by upregulating the expressions of PPARγ and insulin-degrading enzyme in hippocampus of a rat model of AD, demonstrating ginsenoside Rg1 has potential as a therapeutic agent for AD treatment (Quan et al., 2013). Thus, ginsenoside Re and ginsenoside Rg1 may contribute a lot to anti-inflammatory and neuroprotective effects of SBP, as demonstrated here. Supporting this notion, the major components of SBP, e.g. ginsenoside Re, ginsenoside Rg1 and cinnamic acid, could penetrate the blood-brain barrier (Jana et al., 2013; Zhao et al., 2018). Moreover, SBP regulated the expressions of mRNA and protein of apoptotic signaling proteins, e.g. Bcl-2 and Bax. In addition to the previous studies, SBP improved the cognitive impairment of APP/PS1 transgenic mice possibly through neuroprotection, enhancement of cholinergic signal transmission and promote neurogenesis. This notion will be worth to be further verified in more animal models associated with AD.

Drug repositioning or drug repurposing is one of the effective strategies to revisit the existent drugs for novel clinical application. The drug intake by patients might interact and regulate variety of targets, including receptors and kinases. The un-intended interaction between drug and non-therapeutic targets might produce unwanted side effects; however, some side effects, produced by the “off-target,” might be useful to treat other diseases. There are many successful examples of drug repositioning. Sildenafil, commercially known as Viagra, is a well-known example for drug repositioning. Considering that multiple-targeted is one of the characteristics of TCMs, it is reasonable to explore additional therapeutic benefits of TCMs. In this connection, SBP, known in current herbal market, might be potential candidate for drug repositioning. In fact, several studies have tried to demonstrate additional therapeutic effects of SBP. Moreover, the safety and adverse effects of the old drug have been clearly studied. Thus, drug repositioning might greatly reduce the cost and period in drug development process. Our findings have not only indicated that SBP might be a promising candidate as an anti-AD agent, but also shed the light on the strategy of TCM repositioning.

## Conclusion

We provided several lines of evidence to suggest that SBP could inhibit Aβ fibril formation, and the SBP-treated Aβ aggregates were of lower toxicity to neurons, as supported by increase in cell viability. Most encouragingly, the results in our present study demonstrated that SBP could significantly ameliorate the learning and memory impairments, reduce the area of Aβ plaque, suppress secretions of cytokines, and exert anti-apoptotic effects in APP/PS1 transgenic mice model of AD. Our findings extend the knowledge of mechanistic action of SBP in improving the memory impairments of AD, and which support SBP is worthy of further development into pharmaceutical medicines for AD treatment.

## Data Availability Statement

The raw data supporting the conclusions of this article will be made available by the authors, without undue reservation.

## Ethics Statement

The animal study was reviewed and approved by Animal Ethics Committee (Permit Number: HB1808021).

## Author Contributions

W-HH and S-HM performed the experiments. Z-YZ and Y-JX prepared and analyzed the water and ethanol extracts of SBP. MX, RD, and TT-XD performed the HPLC fingerprint analysis and supplied the materials. S-PL, C-SZ, and X-HS designed and analyzed the data. TT-XD and KW-KT organized and supervised the study. All authors contributed to the article and approved the submitted version.

## Funding

This study was supported by Chinese Association of Integrated Traditional and Western Medicine-SHPL Research Fund No. 2018001, Shenzhen Science and Technology Innovation Committee (ZDSYS201707281432317; JCYJ20170413173747440; JCYJ 20180306174903174), China Post-doctoral Science Foundation (2019M653087), Zhongshan Municipal Bureau of Science and Technology (ZSST20SC03); Guangzhou Science and Technology Committee Research Grant (GZSTI16SC02; GZSTI17SC02); Hong Kong RGC Theme-based Research Scheme (T13-605/18-W); Hong Kong Innovation Technology Fund (UIM/340, UIM/385, ITS/500/18FP; TCPD/17-9); TUYF19SC02, PD18SC01, and HMRF18SC06.

## Conflict of Interest

The two authors, C-SZ and X-HS, are employed by the following companies: Shanghai Hutchison Pharmaceuticals Ltd. and Shanghai Engineering Research Center for Innovation of Solid Preparation of TCM.

The remaining authors declare that the research was conducted in the absence of any commercial or financial relationships that could be construed as a potential conflict of interest.

## Supplementary Material

Supplementary Figure 1 HPLC fingerprint of SBP extracts. The chromatographic separation method, both ethanol and water extracts of SBP, i.e. SBPEtOH and SBPwater, was shown in materials and methods section. The identifies of peaks of HPLC fingerprints of SBPEtOH and SBPwater are: (1) benzoic acid from Styrax; (2) cinnamic acid from Styrax and Cinnamomi Cortex; (3) cinnamaldehyde from Styrax and Cinnamomi Cortex; (4) cinobufagin from Bufonis Venenum; (5) recibufogenin from Bufonis Venenum; (6) ethyl cinnamate from Styrax; (7) benzyl benzoate from Styrax; (8) benzyl cinnamate from Styrax; (9) phenethyl cinnamate from Styrax; (10) cinnamyl cinnamate from Styrax; (11) unknown from Styrax; and (12) unknown from Styrax. A typical profile was shown, where n = 4. The SBP extracts, as well as the extracts of various herbs, being used here were same as the one being used in our previous report (Xu et al., 2019).

Supplementary Figure 2 Schematic sketch of mouse brain section. An outline of mouse cross-section showing the brain regions for histochemical analysis. The blue triangle represents the selected cerebral cortex for identification of Aβ plaque, as in **Figure 4**. The red star represents the selected hippocampus for identification of nuclear pyknosis of neurons, as in **Figure 5**. Bar = 1 mm.

## Abbreviations

SBP, Shexiang Baoxin Pill; TCM, traditional Chinese medicine; AD, Alzheimer’s disease; DMEM, Dulbecco’s modified Eagle’s medium; HRP, horse reddish peroxidase; NO, nitric oxide; NOS, nitric oxide synthase; AChE, acetylcholinesterase.
