# Supplementary figures and images for: Shexiang Baoxin Pill, a Traditional Chinese Herbal Formula, Rescues the Cognitive Impairments in APP/PS1 Transgenic Mice
Source: Front Pharmacol. 2020 Jul 14;11:1045. doi: 10.3389/fphar.2020.01045 (PMC7381243; doi:10.3389/fphar.2020.01045)

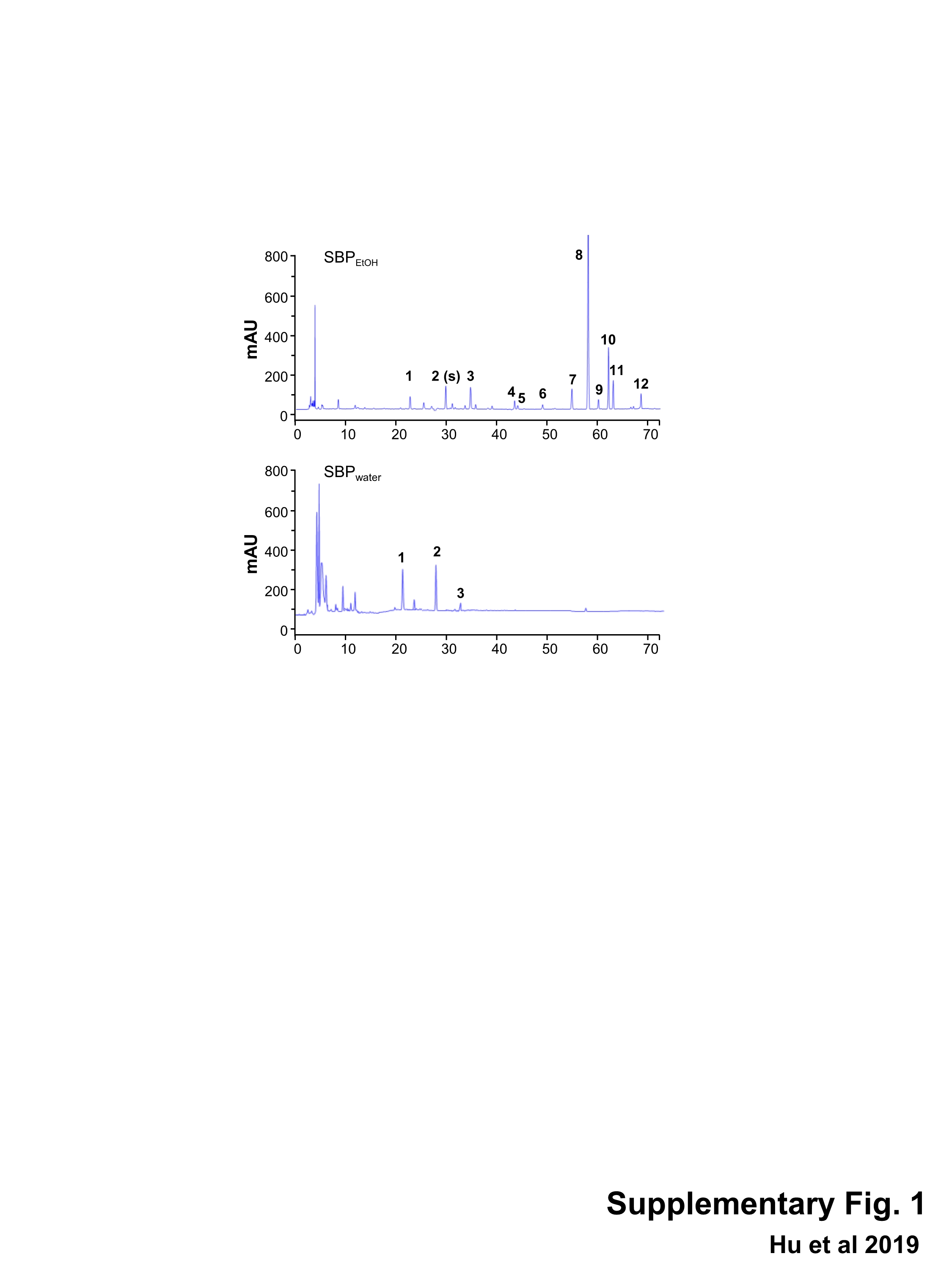

Supplement: Supplementary Figure 1 — HPLC fingerprint of SBP extracts. The chromatographic separation method, both ethanol and water extracts of SBP, i.e. SBPEtOH and SBPwater, was shown in materials and methods section. The identifies of peaks of HPLC fingerprints of SBPEtOH and SBPwater are: (1) benzoic acid from Styrax; (2) cinnamic acid from Styrax and Cinnamomi Cortex; (3) cinnamaldehyde from Styrax and Cinnamomi Cortex; (4) cinobufagin from Bufonis Venenum; (5) recibufogenin from Bufonis Venenum; (6) ethyl cinnamate from Styrax; (7) benzyl benzoate from Styrax; (8) benzyl cinnamate from Styrax; (9) phenethyl cinnamate from Styrax; (10) cinnamyl cinnamate from Styrax; (11) unknown from Styrax; and (12) unknown from Styrax. A typical profile was shown, where n = 4. The SBP extracts, as well as the extracts of various herbs, being used here were same as the one being used in our previous report (Xu et al., 2019). [file Image_1.jpeg]

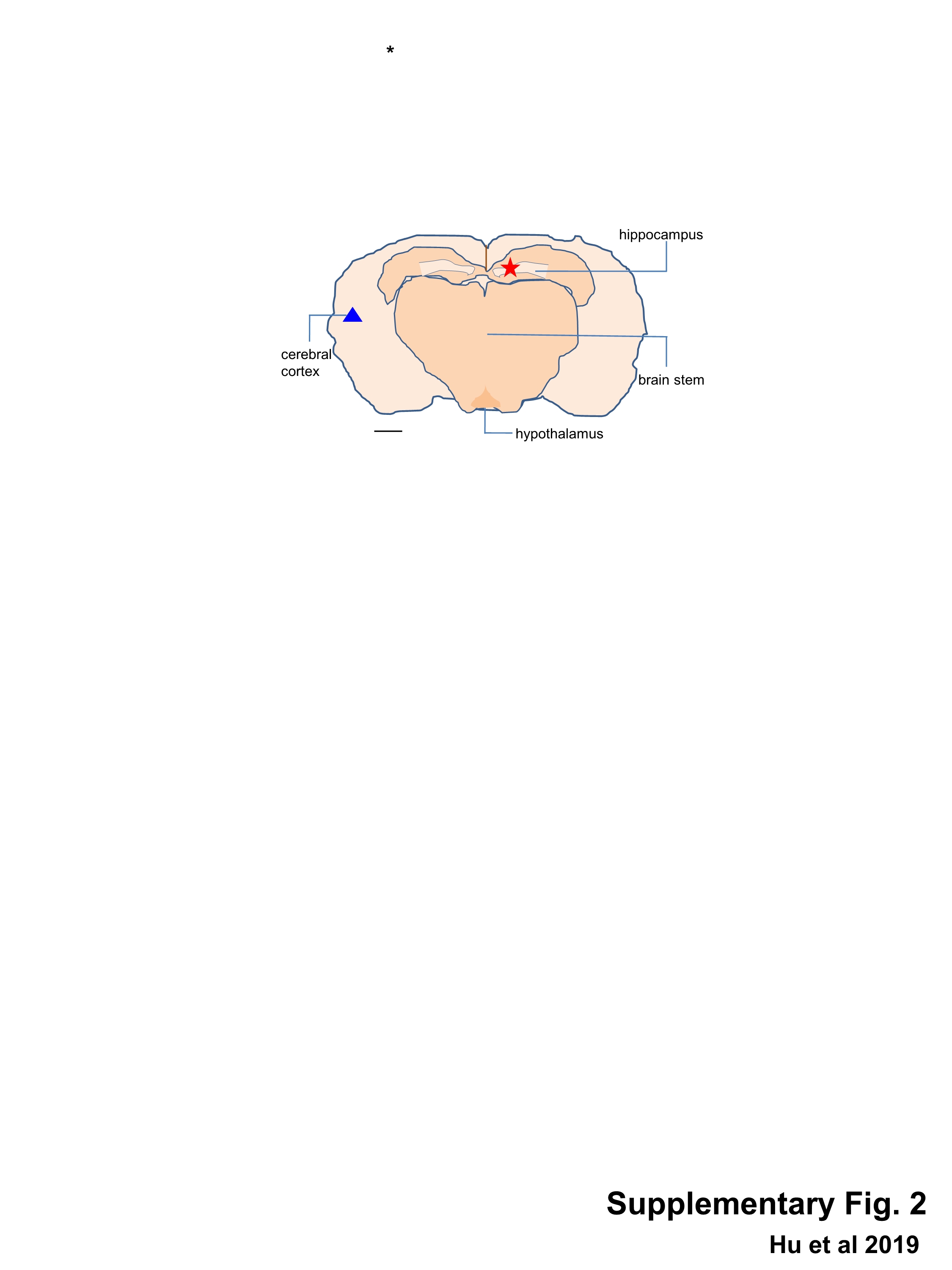

Supplement: Supplementary Figure 2 — Schematic sketch of mouse brain section. An outline of mouse cross-section showing the brain regions for histochemical analysis. The blue triangle represents the selected cerebral cortex for identification of Aβ plaque, as in Figure 4 . The red star represents the selected hippocampus for identification of nuclear pyknosis of neurons, as in Figure 5 . Bar = 1 mm. [file Image_2.jpeg]
